# Draft Genome Sequence of *Pseudomonas plecoglossicida* Strain NB2011, the Causative Agent of White Nodules in Large Yellow Croaker (*Larimichthys crocea*)

**DOI:** 10.1128/genomeA.00586-13

**Published:** 2013-08-08

**Authors:** Zhijuan Mao, Meifang Li, Jigang Chen

**Affiliations:** Biological and Environmental College, Zhejiang Wanli University, Ningbo, Chinaa; Life Science College, Zhejiang Chinese Medical University, Hangzhou, Chinab

## Abstract

We describe the draft genome sequence of *Pseudomonas plecoglossicida* strain NB2011, the causative agent of white nodules in cultured large yellow croaker (*Larimichthys crocea*) in China. The draft genome sequence of the bacterium consists of 5.41 million bp, with a G+C content of 62.8%. A total of 4,952 genes were identified.

## GENOME ANNOUNCEMENT

*Pseudomonas plecoglossicida* is a bacterium that is physically and genetically closely related to *P. putida* and has been assigned as a member of the *P. putida* group (1). The bacterium was first identified in cultured ayu (*Plecoglossus altivelis*) with bacterial hemorrhagic ascites in 2000 (2); recently, infections in rainbow trout (*Oncorhynchus mykiss*) and large yellow croaker (*Larimichthys crocea*) were also reported (3, 4). In large yellow croaker, the infection leads to the development of white nodules in the spleen, kidney, and liver of the diseased fish and results in high mortality (4, 5). The causative bacterium, strain NB2011, was preliminarily identified as *P. putida* by physical and biochemical profiles and 16S rRNA sequencing (5), but the strain was reclassified as *P. plecoglossidica* based on a *gyrB* sequence revealed by genome sequencing (6).

Here, we report the draft genome sequence of *P. plecoglossicida* strain NB2011, isolated from a diseased large yellow croaker with typical symptoms of white nodules in the internal organs (5). The sequencing was performed by Illumina Hiseq 2000. The data generated 7,361,580 reads totaling 3.68 Gb. The gaps were closed by specific PCR and Sanger sequencing. Genome sequences were assembled by SOAPdenovo (7), resulting in 87 contigs (>500 bp in size) with an *N*_50_ length of 159,696 bp, and a total length of about 5.4 Mb. The genome sequence was annotated using the databases RAST (8) and PGAAP (http://www.ncbi.nlm.nih.gov/genome/annotation_prok/). The pathogenicity islands (PAIs) were predicted by PIPS (9).

The NB2011 draft genome sequence has a total of 5,413,333 bp, with a G+C content of 62.8%. It contains 4,952 genes, including 4,883 predicted coding sequences (CDS), 66 tRNA genes, and one copy each of 16S rRNA, 23S rRNA, and 5S rRNA genes. The genome sequences reveal many virulence mechanisms similar to those of other pathogenic pseudomonads, including the type III and type VI secretion system, type IV pilus, and multiple flagellins. A 24-kb gene cluster encoding a putative type III secretion system is presented in a genome island showing characteristics of PAI, and most of the functional proteins were identified. Two gene clusters involved in flagellar protein synthesis were annotated; the one with a length of about 32 kb was also arranged in a PAI-like genome island and the predicted amino acid sequences showed lower similarity (60 to 80%) to corresponding flagellar proteins of other Gram-negative bacteria. Pseudomonads usually express various enzymes, including catalases, peroxidases, and superoxide dismutases (SOD), for defense against reactive oxygen species (ROS). The strain is predicted to encode four catalases, three SODs, and three glutathione peroxidases, which may also be related to intracellular survival in macrophages (10, 11). Nine CDS encoding drug-resistance transporters and small multidrug-resistance proteins were annotated; these may be related to the intrinsic resistance of this strain to many antibiotics/drugs. However, no evidence was found in this strain for putative functions of pseudomonad extracellular pathogenicity factors, such as exotoxin A or phospholipase C.

### Nucleotide sequence accession number.

The genome sequence of *P. plecoglossicida* NB2011 has been deposited in the GenBank database under accession number ASJX00000000. The version described in this paper is the first version.
